# Low Aerobic Capacity Accelerates Lipid Accumulation and Metabolic Abnormalities Caused by High-Fat Diet-Induced Obesity in Postpartum Mice

**DOI:** 10.3390/nu14183746

**Published:** 2022-09-10

**Authors:** Mon-Chien Lee, Yi-Ju Hsu, Hsin-Ching Sung, Ya-Ting Wen, Li Wei, Chi-Chang Huang

**Affiliations:** 1 Graduate Institute of Sports Science, National Taiwan Sport University, Taoyuan 333325, Taiwan; 2 Department of Anatomy, College of Medicine, Chang Gung University, Taoyuan 333323, Taiwan; 3 Aesthetic Medical Center, Department of Dermatology, Chang Gung Memorial Hospital, Taoyuan 333423, Taiwan; 4 Division of Neurosurgery, Department of Surgery, Wan Fang Hospital, Taipei Medical University, Taipei 116081, Taiwan; 5 Graduate Institute of Injury Prevention and Control, Taipei Medical University, Taipei 110301, Taiwan; 6 Taipei Neuroscience Institute, Taipei Medical University, Taipei 110301, Taiwan

**Keywords:** postpartum, exercise capacity, high-fat diet, obesity, metabolic disorders

## Abstract

Women during pregnancy and postpartum show high rates of obesity and metabolic diseases, especially women with excessive caloric intake. In the past, it was proved that individuals with high intrinsic aerobic exercise capacities showed higher lipid metabolism and lower fat production than those with low intrinsic aerobic exercise capacities. The purpose of this study was to determine whether mice with the low-fitness phenotype (LAEC) were more likely to develop metabolic abnormalities and obesity under dietary induction after delivery, and if mice with a high-fitness phenotype (HAEC) had a protective mechanism. After parturition and weaning, postpartum Institute of Cancer Research (ICR) mice received dietary induction for 12 weeks and were divided into four groups (*n* = 8 per group): high-exercise capacity postpartum mice with a normal chow diet (HAEC-ND); high-exercise capacity postpartum mice with a high-fat diet (HAEC-HFD); low-exercise capacity postpartum mice with a normal chow diet (LAEC-ND); and low-exercise capacity postpartum mice with a high-fat diet (LAEC-HFD). Obesity caused by a high-fat diet led to decreased exercise performance (*p* < 0.05). Although there were significant differences in body posture under congenital conditions, the LAEC mice gained more weight and body fat after high-fat-diet intake (*p* < 0.05). Compared with HAEC-HFD, LAEC-HFD significantly increased blood lipids, such as total cholesterol (TC), triacylglycerol (TG), low-density lipoprotein (LDL) and other parameters (*p* < 0.05), and the content of TG in the liver, as well as inducing poor glucose tolerance (*p* < 0.05). In addition, after HFD intake, excessive energy significantly increased glycogen storage (*p* < 0.05), but the LAEC mice showed significantly lower muscle glycogen storage (*p* < 0.05). In conclusion, although we observed significant differences in intrinsic exercise capacity, and body posture and metabolic ability were also different, high-fat-diet intake caused weight gain and a risk of metabolic disorders, especially in postpartum low-fitness mice. However, HAEC mice still showed better lipid metabolism and protection mechanisms. Conversely, LAEC mice might accumulate more fat and develop metabolic diseases compared with their normal rodent chow diet (ND) control counterparts.

## 1. Introduction

The postpartum period is called the “fourth stage of labor”, which is the period when the maternal physiological and metabolic changes return to a non-pregnant state after the pregnant woman gives birth [1]. Postpartum can be divided into three different but continuous phases, including: (1) the initial or acute phase of 6–12 h postpartum; (2) the subacute phase of maintenance to 2–6 weeks postpartum; and (3) the delayed postpartum period from 6 weeks to 6 months [2]. Just like pregnancy, they are two critical periods in a woman’s life that have an impact on the body. Gaining proper weight during pregnancy has direct and long-term health effects on offspring and mothers. However, excessive weight gain during pregnancy (EGWG) may cause long-term obesity after childbirth [3]. Previous studies have shown that as many as 20% of women maintain a weight of more than 4 kg in the first year after childbirth [4], even for up to 15 years [5]. In addition, postpartum weight maintenance might be related to reduced healthy eating behavior, decreased physical activity, lack of sleep time, and postpartum depression [6], which might increase postpartum women’s risk of chronic diseases such as cardiovascular disease, type II diabetes, osteoarthritis, and certain cancers [7]. Therefore, postpartum recovery is essential to avoid the effects of weight retention.

Aerobic exercise can increase human endurance, enhance heart and cardiorespiratory ability, and can promote physical health and exercise performance [8]. On the other hand, it can also reduce and prevent the risk of metabolic diseases and mortality such as dyslipidemia, diabetes, hypertension, and obesity [9]. However, aerobic capacity is affected by genetic and environmental factors, and individuals with different genetic characteristics show different responses to exercise [10]. In addition, there are big differences in energy utilization and metabolism [11]. Previous studies have found that artificially selected high or low endurance running rats are different in their susceptibility to health, ageing, prolonged life cycle, and chronic disease risk factors including metabolic syndrome and cardiovascular complications [12]. In previous studies, after 10 generations of breeding, rats with low exercise capacity were significantly heavier than those with high exercise capacity. In addition, Compared with LCR rats, HCR rats had resting energy expenditure, internal body temperature, skeletal muscle fever and other thermogenic phenotypes during exercise [11], and could significantly improve free fatty acids, visceral fat, triglycerides, and mitochondrial-related expressed proteins [13]. All these unique cellular and physiological characteristics could prevent HCR rats from diet-induced obesity, insulin resistance, and liver steatosis [14]. Low aerobic exercise capacity and oxidative metabolism are associated with many chronic diseases such as insulin resistance, metabolic syndrome, type 2 diabetes, and cardiovascular disease [15], indicating that low fitness during exercise leads to a reduction in mitochondrial-synthesis-related transcription factors in vivo and the reduction in oxidase in skeletal muscle. These defects are also related to cardiovascular adaptability and related metabolic diseases [16,17].

Due to the excessive intake of high-fat diets, women after pregnancy are at a higher risk of metabolic-syndrome-related diseases. However, high intrinsic exercise capacity and aerobic exercise were shown to have protective effects on visceral fat, free fatty acids, and triglycerides [18]. In this study, we screened mice with high and low aerobic capacity using our well-established swimming endurance exercise platform and were fed either normal or high-fat diets with the similar of calories after pregnancy to explore the effect of different intrinsic exercise capacities on blood biochemistry, exercise capacity, and pathology after high-fat diet intake.

## 2. Materials and Methods

### 2.1. Materials

We developed high or low aerobic exercise capacity (AEC) mouse (HAEC or LAEC, respectively) models displaying high and low intrinsic aerobic capacity as previously described [19]. For this study, 32 randomly cycling female Institute for Cancer Research (ICR) HAEC and LAEC mice (Generation 9) after production were housed in the animal facility at National Taiwan Sport University (NTSU) with an environment maintained with a constant photoperiod, humidity, and temperature (12 h light/12 h dark cycle, 50–60%, and 24 ± 2 °C, respectively). Distilled water and standard laboratory chow diet (No. 5001; PMI Nutrition International, Brentwood, MO, USA) were provided ad libitum. The Institutional Animal Care and Use Committee (IACUC) of NTSU approved all animal experiments in this study, and the study conformed to the guidelines of the IACUC-10622 protocol approved by the IACUC ethics committee.

### 2.2. Animals and Experimental Design

After three weeks’ breastfeeding, 32 postpartum mice (14 weeks old) were randomized either to remain on normal rodent chow diet (ND) (containing 3.36 kcal/g with 28.5% protein, 13.4% fat, and 58.1% carbohydrates) or were switched to a high-fat diet (HFD), which was refer to the previous description and home-made [20] (containing 35% *w*/*w*, lard oil, 15% *w*/*w* fructose, 0.2% cholesterol, and 49.8% *w*/*w* standard chow, for 5.13 kcal/g with 12.5% protein, 36.7% fat, and 38.3% as carbohydrates), then generating into four groups: HAEC-ND (*n* = 8), HAEC-HFD (*n* = 8), LAEC-ND (*n* = 8), and LAEC-HFD (*n* = 8) for 12 weeks. Food intake and water consumption were monitored daily, and body weight was recorded weekly.

### 2.3. Exercise Endurance Performance Test

After 12 weeks’ dietary intervention, physical function was characterized by measuring running time and distance using a motorized 10-lane treadmill (MK680C, Muromachi Kikai Co. Ltd., Tokyo, Japan), as previously described [21]. All the mice were acclimated to the treadmill for three consecutive days for 5 min at a speed of 10 m/min at a 5% grade. On the test day, all the mice were run on the treadmill at an initial speed of 15 m/min and grade 15% for 2 min, and then in 2 min intervals, the speed was increased by 3 m/min until exhaustion [22]. Exhaustion was defined as the inability of the mouse to remain on the treadmill despite an electric shock stimulus and mechanical prodding for a period of 5 s.

### 2.4. Oral Glucose Tolerance Test (OGTT)

After twelve weeks on experimental diets, mice were placed in cages without feed and subjected to 16 h fasted for oral glucose tolerance tests (OGTTs). Glucose was measured in whole blood taken from the tail vein with a Bayer Contour glucometer and glucose test strips (Bayer, Barmen, Germany). Glucose was administered by oral gavage (2 g/kg body weight (BW)), and the blood glucose concentration was measured 15, 30, 60, and 120 min after glucose administration. The blood glucose incremental area under the curve (AUC, mg/dL/h) was calculated as the AUC above the baseline value, i.e., the 16 h fasted blood glucose, by applying the trapezoid rule to a plot of group mean blood glucose concentrations versus times of measurements [23,24].

### 2.5. Tissue Sample Preparation and Histopathology

At the end of the experiments, all mice were killed by 95% CO_2_ asphyxiation, and blood was withdrawn by cardiac puncture after a 16 h fast. The target organs and tissues collected included the heart, liver, lungs, kidneys, muscle tissue, ovarian fat pad (OFP), mesenteric fat pad, perirenal fat pad, and brown adipose tissue (BAT). Organs and tissues were carefully excised and rinsed in saline solution, and then blotted dry with a kimwipe. The whole weight and the specific tissue weight (%) relative to individual body weight were recorded and calculated. The visceral organs preserved in 10% formalin were trimmed and embedded in paraffin for tissue sections in 4 μm thick slices. Tissue sections were further stained with hematoxylin and eosin (H&E) and examined under a light microscope equipped with a (charge-coupled device) CCD camera (BX-51, Olympus, Tokyo, Japan) by a veterinary pathologist.

### 2.6. Hepatic Lipid Profile and Glycogen Assay

Parts of the liver and muscle tissues were stored in liquid nitrogen and glycogen content were analyzed as previously described [21].

The liver tissue (350 mg) was homogenized in 2 mL of cold buffer (i.e., chloroform/isopropanol/NP40 = 7:11:0.1), and the sample was centrifuged at 10,000× *g* at 4 °C for 10 min for the analysis. For this test, 100 μL of the sample was added to the wells, and the plate cover was removed by adding 50 μL of the prepared assay mixture to all the wells to initiate the reaction. The TC concentration was determined using a cholesterol fluorescence assay kit (product number: 10007640, Cayman Chemical Company, Ann Arbor, MI, USA).

The remixed liver TG was measured using a triacylglycerol absorbance assay kit (product number: 10010303, Cayman Chemical Company, Ann Arbor, MI, USA). After removing the supernatant, we remixed the sample with dilution buffer (50 mM sodium phosphate, pH 7.2). We mixed the liver extract with reagent buffer thoroughly and measured the absorbance value at 560 nm optical density (OD).

### 2.7. Measurement of Biochemical Parameters

After the 12 weeks HFD experiments, blood samples were immediately collected from the submandibular duct of each mouse from the treated groups after a 16 h fast and centrifuged at 1500× *g* and 4 °C for 10 min for serum preparation. Serum was collected by centrifugation, and levels of total cholesterol (TC), triacylglycerol (TG), high-density lipoprotein (HDL), low-density lipoprotein (LDL), and glucose (GLU) were assessed using an auto-analyzer (Hitachi 7060, Hitachi, Ltd., Tokyo, Japan).

### 2.8. Statistical Analysis

The data are represented as mean ± standard error of the mean (SEM). Two-way ANOVA was used to assess the effects of the intrinsic exercise capacity and high-fat diet on general mouse characteristics, including body weight, organ weight, biochemical values, swimming exhaustion times, and grip strength. Tukey’s honestly significant different (HSD) test was used to compare individual means among treatment groups. *p* < 0.05 was considered statistically significant. Statistical analyses were performed using SAS v9.0 (SAS, Cary, NC, USA).

## 3. Results

### 3.1. Animal Characteristics and Phenotype

Descriptive data for the HAEC and LAEC mice are presented in Table 1. The swimming capacity assessed at six weeks of age was over 38-fold greater in the HAEC mice, thus demonstrating the marked difference in aerobic phenotype between these strains due to the selective breeding paradigm [19].

**Figure 1:**
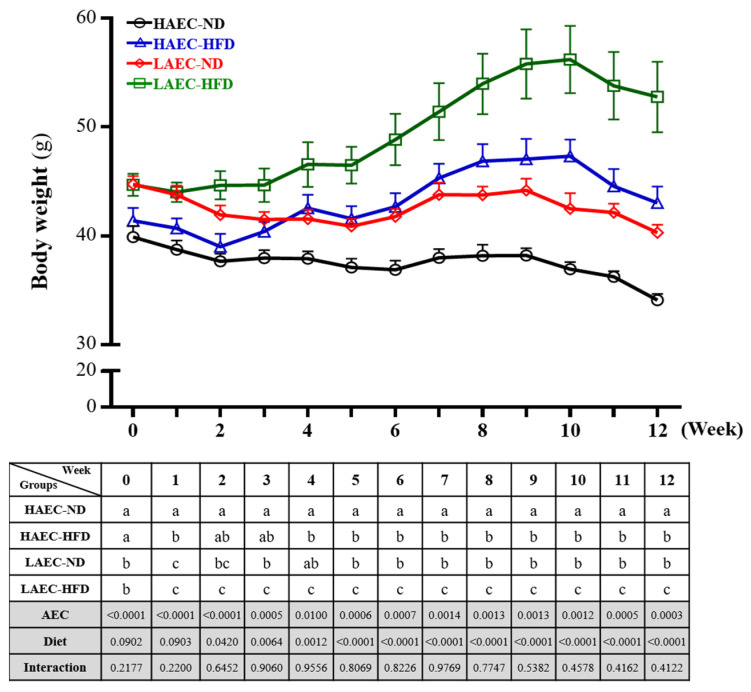
Effects of 12 weeks of high-fat diet (HFD) feeding of high aerobic exercise capacity (HAEC) or low aerobic exercise capacity (LAEC) postpartum mice on body weight. Data are mean ± standard error of the mean (SEM) for *n* = 8 mice per group. Columns with different superscript letters (a, b, c) in table are significantly different between the group at *p* < 0.05.

Over time, the body weight of mice steadily increased (Figure 1). The LAEC-HFD group was significantly heavier than the other three groups (*p* < 0.05). The main effect of AEC was a significant decrease in body weight (*p* < 0.0015) and the main effect of HFD was a significant increase in body weight (*p* < 0.0001).

Food, water, and energy intake were recorded on a daily basis, as presented in Table 1. To investigate the effects of and changes in the HFD on the body, the ND and HFD with similar calorie were fed in HAEC or LAEC groups, respectively. Regardless, LAEC mice consuming the ND was significantly higher than that of HAEC (18.22 ± 0.24 and 20.92 ± 0.32 kcal/day, respectively; *p* < 0.05), and the HFD results were similar (17.67 ± 0.42 and 20.57 ± 0.34 kcal/day, respectively; *p* < 0.05). Therefore, diet intake differed between groups (*p* < 0.05). No difference between HAEC and LAEC was observed in consumed water with the normal diet (9.35 ± 0.14 and 9.10 ± 0.34 mL/day, respectively) or the HFD (4.42 ± 0.23 and 4.09 ± 0.08 mL/day, respectively), but ND groups consumed significantly greater than HFD groups (*p* < 0.0001).

The calculation of energy efficiency was based on (final weight-initial weight)/experimental total calories × 100%, which was equivalent to the effect of 1 kcal on the weight change rate of HAEC or LAEC mice. We found that after the postpartum and breast-feeding, the 12 weeks ND intake of the HAEC and LAEC groups were gradually decreased, and there was no significant difference between the two groups. However, in terms of HFD intake, the average weight growth rate of the LAEC group (0.13 ± 0.27) per 1 kcal intake was significantly increased by 4-fold (*p* = 0.0087) compared with the HAEC group (0.52 ± 0.51).

### 3.2. Tissue Weight and Morphological Examination

The organ weights for intervention and control animals can provide information about the health statuses of test mice. As shown in Table 2, in HAEC and LAEC mice, the liver weight of mice fed the HFD was significantly greater than those fed ND (*p* < 0.05), and the relative liver weights were similar. The main effect of AEC was significant decreases in liver weight (*p* = 0.0251) and relative liver weight (*p* = 0.0249); the main effect of the HFD was significant increases in liver weight (*p* = 0.0011) and relative liver weight (*p* = 0.0010). The kidney weight in LAEC mice in ND or HFD groups was significantly greater than that of HAEC mice (*p <* 0.05), and the relative kidney weights were similar. The main effect of AEC was significant decreases in kidney weight (*p* = 0.0003) and relative kidney weight (*p* = 0.0057); the main effect of the HFD was significant decreases in absolute kidney weight (*p* = 0.0057) and relative kidney weight (*p* = 0.0060). In HAEC and LAEC mice, the weights of perirenal fat, mesenteric fat, and ovarian fat in the HFD intake group were significantly higher than that in the ND-intake group (*p* < 0.05), which were similar to the relative perirenal fat, mesenteric fat, and ovarian fat weights. The LAEC-HFD group’s mesenteric fat was significant heavier than that of the other three groups (*p* < 0.05). The main effect of AEC was not significant, but the main effect of the HFD was significant increases in weight (*p* < 0.0001) and relative weight (*p* < 0.0001). The muscle weight in the HFD groups was significantly higher than those fed ND in both HAEC and LAEC mice (*p* < 0.05), and the relative muscle weight were same. The main effect of AEC was not significant, but the main effect of the HFD was significant increases in muscle weight (*p* = 0.0006) and relative muscle weight (*p* = 0.0005). The heart weight in HAEC or LAEC mice did not differ between ND or HFD groups. The main effect of AEC was significant decreases in heart weight (*p =* 0.0131) and relative heart weight (*p* = 0.0144), but the main effect of the HFD was not significant. The lung and BAT weights were no different between ND or HFD groups in HAEC or LAEC mice, but lung and BAT weights were significantly lower in the HAEC than in the LAEC mice (*p* < 0.05); the findings for relative lung and BAT weights were similar. The main effect of AEC was significant decreases lung and BAT weights (*p* < 0.0001 and *p* = 0.0057, respectively) and relative lung and BAT weights (*p* < 0.0001 and *p* = 0.0144, respectively), but the main effect of the HFD was not significant. Although of the above weights may be affected by AEC or HFD, we found no significant interaction between the two in the above-mentioned tissues.

### 3.3. Exercise Endurance Performance Test with HAEC and LAEC Postpartum Mice

**Figure 2:**
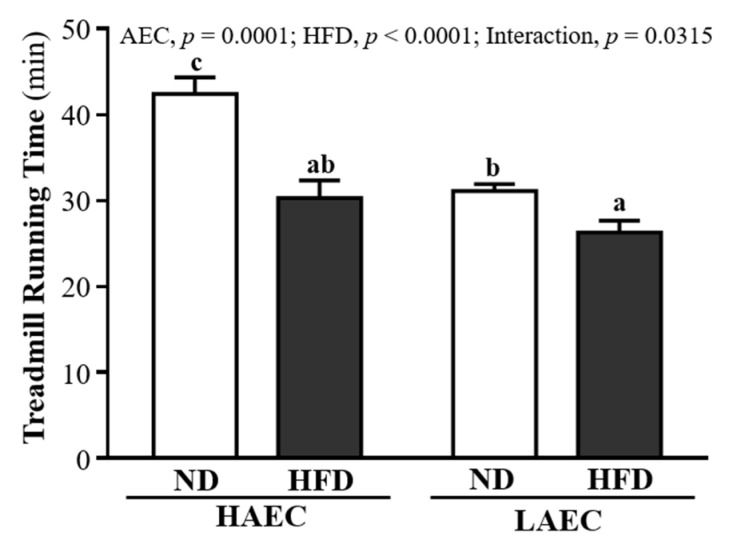
Effects of 12 weeks’ HFD feeding of HAEC or LAEC postpartum mice on endurance treadmill running test. Data are mean ± SEM for *n* = 8 mice per group. Columns with different superscript letters (a, b, c) are significantly different at *p* < 0.05.

One of the physical performance tests was the exhaustive treadmill running exercise (Figure 2). HAEC-ND mice endured longer (42.40 ± 1.94 min) than HAEC-HFD mice (30.31 ± 2.04 min), LAEC-ND mice (31.11 ± 0.83 min), and LAEC-HFD mice (26.28 ± 1.40 min), by 1.40-fold (*p* < 0.0001), 1.36-fold (*p* < 0.0001), and 1.61-fold (*p* < 0.0001), respectively. For both the swimming and treadmill running tests, the intrinsic aerobic capacity did not change: the HAEC groups still endured longer than the LAEC groups, but HFD intake reduced the endurance performance compared with ND intake. The main effect of AEC was increased treadmill running time (*p* = 0.0001) and the main effect of HFD was decreased treadmill running time (*p* < 0.0001).

### 3.4. OGTT Glucose Level of HAEC and LAEC Postpartum Mice

**Figure 3:**
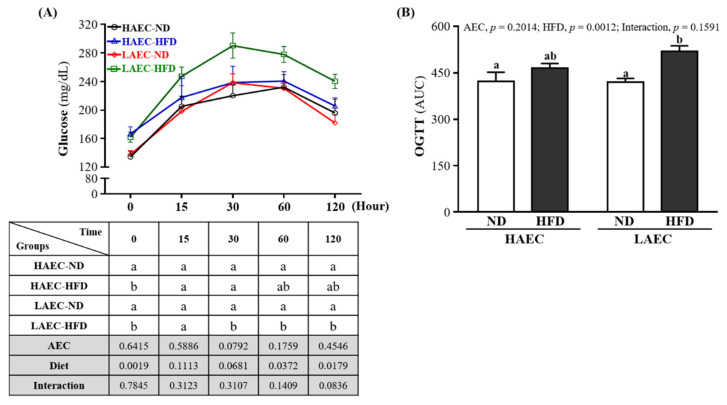
Effects of 12 weeks’ HFD feeding of HAEC or LAEC postpartum mice on: (**A**) oral glucose tolerance test (OGTT) curve; and (**B**) area under the curve. Data are mean ± SEM for *n* = 8 mice per group. Columns with different superscript letters (a, b) are significantly different between groups at *p* < 0.05.

As obesity, visceral adiposity, and hepatic steatosis are associated with impaired glucose and insulin homeostasis, we subjected the mice to 16 h fasted OGTT after 12 weeks of HFD feeding. At baseline, HFD mice had significantly higher fasting blood glucose than ND mice within the HAEC mice (ND 134.38 ± 21.69 and HFD 166.63 ± 27.61 mg/dL, *p* = 0.0155) and the LAEC mice (ND 137.75 ± 15.09 and HFD,161.88 ± 19.88 mg/dL, *p* = 0.0310). After glucose load, we found no difference in the glucose concentration between HAEC-ND and HAEC-HFD groups over time, but LAEC-HFD mice showed significantly higher glucose concentrations than LAEC-ND at 30, 60, and 120 min (*p* < 0.05, Figure 3A). As demonstrated by the area under the curve (Figure 3B), there was no different between ND and HFD within HAEC groups. However, the glucose concentration of the LAEC-HFD group (519.60 ± 51.67 mg/dL) was significantly higher than that of the LAEC-ND group (420.06 ± 33.90 mg/dL) by 1.24-fold (*p* = 0.0017). The main effects of the HFD were increasing the glucose level and decreasing glucose tolerance (*p* < 0.05).

### 3.5. Glycogen of HAEC and LAEC Postpartum Mice

**Figure 4:**
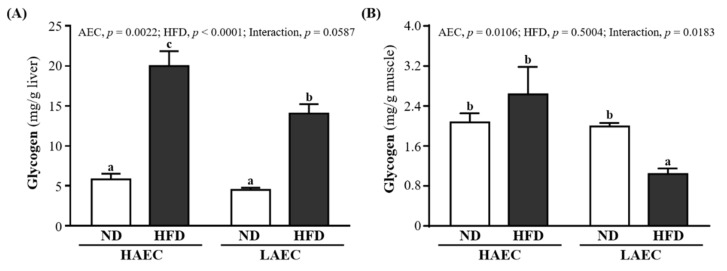
Effects of 12 weeks’ HFD feeding of HAEC or LAEC postpartum mice on: (**A**) liver; and (**B**) muscle glycogen contents. Data are mean ± SEM for *n* = 8 mice per group. Columns with different superscript letters (a, b, c) are significantly different between groups at *p* < 0.05.

The glycogen contents in the liver and skeletal muscles of the mice are shown in Figure 4. The liver glycogen contents of the HFD groups were significantly higher than those of ND groups by 3.44-fold (*p* < 0.0001) in HAEC and 3.12-fold in LAEC. However, the muscle glycogen contents of ND- and HFD-fed mice were no different in HAEC mice but were significantly higher in LAEC-ND mice than in LAEC-HFD mice by 1.92-fold (*p* = 0.0309, Figure 4A). The main effect of AEC was increased liver (*p* = 0.0022) and muscle (*p* = 0.0106) glycogen. The main significant effect of the HFD was showed a significant interaction effect on the liver glycogen index (*p* < 0.0001), but only muscle glycogen showed a significant interaction effect (*p* = 0.0183, Figure 4B).

### 3.6. Hepatic TG and TC Levels of HAEC and LAEC Postpartum Mice

**Figure 5:**
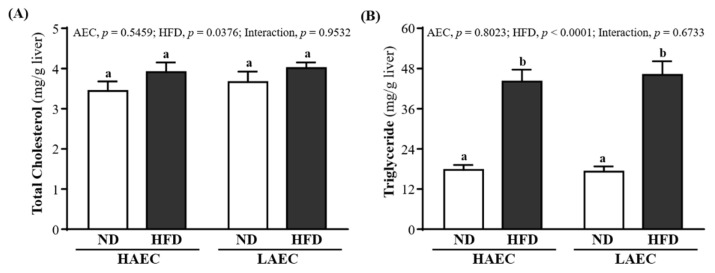
Effects of 12 weeks’ HFD feeding of HAEC or LAEC postpartum mice on: (**A**) hepatic TC; and (**B**) TG levels. Data are mean ± SEM for *n* = 8 mice per group. Columns with different superscript letters (a, b) are significantly different between groups at *p* < 0.05.

At the end of the experiment, liver TC content was 3.44 ± 0.24, 3.91 ± 0.24, 3.66 ± 0.26, and 4.01 ± 0.14 (mg/g liver) for HAEC-ND, HAEC-HFD, LAEC-ND, and LAEC-HFD groups, respectively; there were no significant differences between groups (Figure 5A). The liver TG level contents were 17.72 ± 1.48, 44.11 ± 3.54, 17.21 ± 1.57, and 46.08 ± 4.06 mg/g liver for HAEC-ND, HAEC-HFD, LAEC-ND, and LAEC-HFD groups, respectively (Figure 5B). That of the ND group was significantly lower than that of the HFD group in the HAEC mice by 59.84% (*p* < 0.0001) and LAEC by 62.65% (*p* < 0.0001). The main effect of HFD was increasing liver TC and TG levels (*p* = 0.0376 and *p* < 0.0001, respectively).

### 3.7. Blood Lipid Index of HAEC and LAEC Postpartum Mice

**Figure 6:**
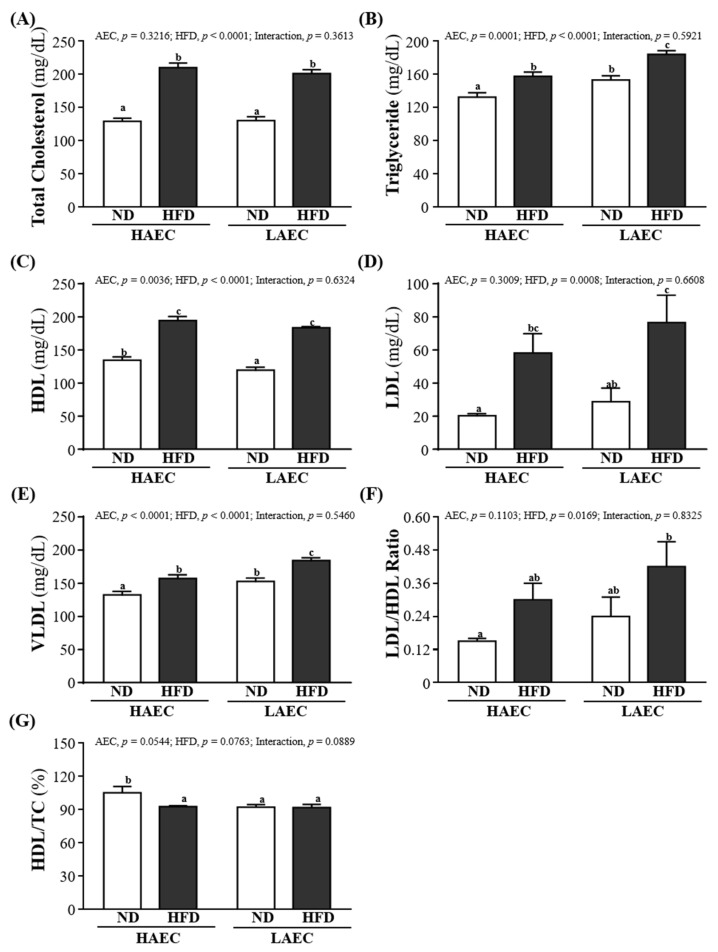
Effects of 12 weeks’ HFD feeding of HAEC or LAEC postpartum mice on blood lipid parameters: (**A**) total cholesterol (TC); (**B**) triglyceride (TG); (**C**) high-density lipoprotein (HDL); (**D**) low-density lipoprotein (LDL); (**E**) very low-density lipoprotein; (**F**) LDL/HDL ratio; and (**G**) HDL/TC ratio. Data are mean ± SEM for *n* = 8 mice per group. Columns with different superscript letters (a, b, c) are significantly different between groups at *p* < 0.05.

For obesity, blood lipid indexes, such as TC, TG, HDL, LDL, etc., are usually detected as a basis for possible metabolic or cardiovascular diseases. As shown in Figure 6A–D, in both HAEC and LAEC mice, the serum TC, TG, HDL, and LDL levels in the HFD-fed groups were significantly higher than in the ND-fed groups (*p* < 0.05): the serum TG and LDL levels in LAEC-HFD were the worst compared with the other three groups. The main effect of AEC was positive on TG and HDL levels; the main effect of HFD was worsening of the TC, TG, HDL, and LDL levels. Other lipid-related indicators can be used to determine cardiovascular disease such as V-LDL, LDL/HDL ratio, HDL/TC, etc. As shown in Figure 6E, the VLDL level in the LAEC mice consuming the ND was significantly lower than that of the HAEC mice by 13.40% (26.50 ± 1.09 and 30.60 ± 1.02 mg/dL, respectively, *p* = 0.0073); the HFD result was similar at 14.46% (31.50 ± 1.02 and 36.83 ± 0.87 mg/dL, respectively, *p* = 0.0001). The LDL/HDL ratio was no different between ND or HFD and HAEC or LAEC, but that of the HAEC-ND mice (0.15 ± 0.01) was significantly lower than that of the LAEC-HFD mice (0.42 ± 0.09) by 63.80% (*p* = 0.0061; Figure 6F). Figure 6G shows that the levels of HDL/TC in the HAEC-ND group were significantly higher by 11.79% (*p* = 0.0166), 12.34% (*p* = 0.0126), and 12.60% (*p* = 0.0110) than in the HAEC-HFD, LAEC-ND, and LAEC-HFD groups, respectively. The main effect of AEC was decreasing of the VLDL level (*p* < 0.0001). The main effect of the HFD was an increase of VLDL and LDL/HDL ratios (*p* < 0.0001 and *p* = 0.0169, respectively).

### 3.8. Tissue Histology of HAEC and LAEC Postpartum Mice

**Figure 7:**
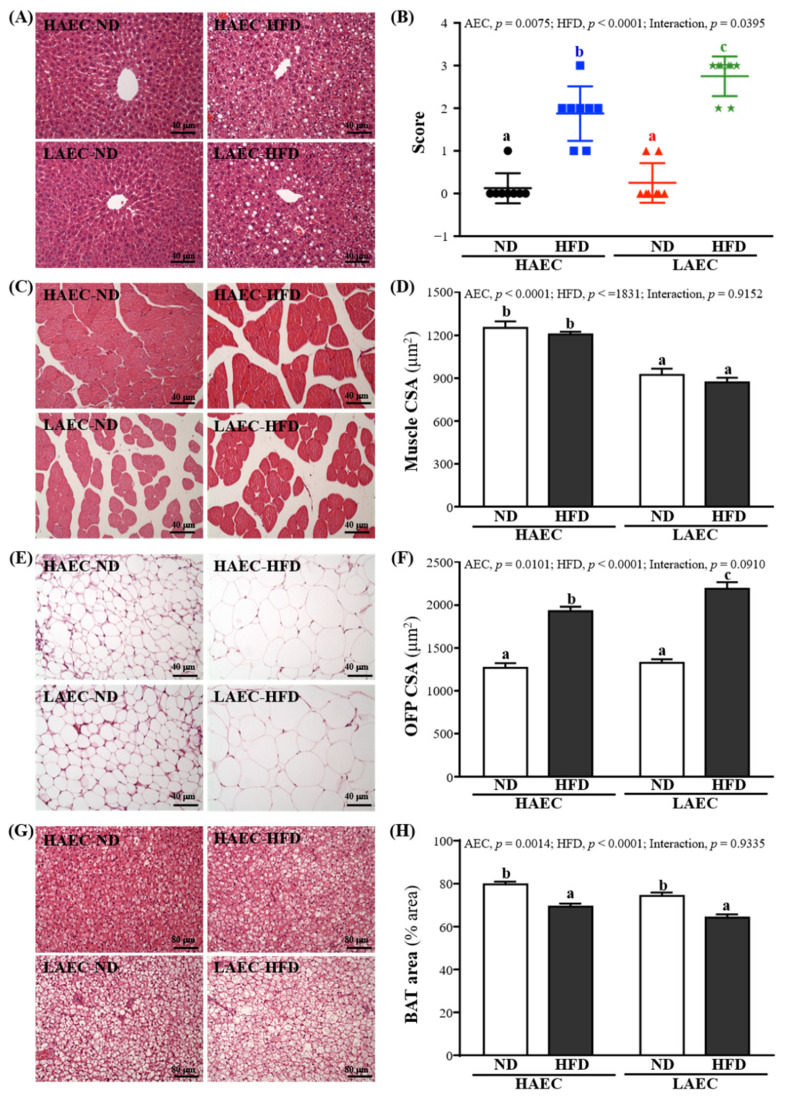
Effects of 12 weeks’ HFD feeding of HAEC or LAEC postpartum mice on histomorphologic features of the: (**A**) liver; (**B**) hepatocyte ballooning; (**C**) muscle; (**D**) muscle fiber CSA; (**E**) OFP; (**F**) OFP fiber CSA; (**G**) BAT; and (**H)** BAT % area. Columns with different superscript letters (a, b, c) are significantly different between groups at *p* < 0.05. Specimens were photographed under a light microscope with hematoxylin and eosin (H&E) stain. CSA, cross section area; OFP, ovarian fat pad; BAT, brown adipose tissue.

In H&E staining of liver sections (Figure 7), HAEC-ND and LAEC-ND normal liver cells were arranged in proper order, with no lipid droplets (microvesicular fat) in lobes. However, HAEC-HFD and LAEC-HFD mice showed an accumulation of lipid droplets in the liver. Thus, HAEC and LAEC mice fed the HFD for 12 weeks showed increased accumulation of microvesicular fat and fatty liver (Figure 7A,B). H&E staining of muscle sections showed more myofibrils and muscle bundles in HAEC groups than in LAEC groups, but both HAEC and LAEC mice fed the HFD for 12 weeks showed decreased myofibrils (Figure 7C,D). OFP showed bigger fat vacuoles in HFD groups than in ND groups. Large fat droplets accumulated freely in the BAT of HAEC groups compared with the LAEC groups; HFD groups accumulated more large fat droplets than ND groups (Figure 7E,F).

## 4. Discussion

Exercise capacity depends not only on the effect of training, but also on inherent genetic factors, which can effectively promote health and reduce the risk of metabolic diseases. However, the underlying mechanisms through which aerobic capacity impact whole-body and tissue-specific energy metabolism and thus predisposition for metabolic disease states remain unclear [18]. In this study, we selected postpartum mice in a high-risk group of metabolic diseases and induced them through a high-fat diet to explore the protective ability and roles of aerobic capacity under different intrinsic aerobic exercise capacities. The results confirmed that although excessive intake of a high-fat diet can affect exercise performance, HAEC is more effective than LAEC in preventing metabolic disorders, fat accumulation, and other metabolic diseases.

We screened intrinsic aerobic capacity through weight-bearing swimming and used artificial propagation to establish the mouse model of intrinsic aerobic capacity. Compared with LAEC, the endurance running time of HAEC significantly increased 38-fold. In addition to significant differences in exercise capacity, the organs were also affected by physiological functions and metabolism. A past study had found that compared with HCR, LCR was more prone to cardiomyocyte hypertrophy and cardiac fibrosis [25]. In addition, since many studies have shown that LCR has a higher body weight than HCR, the volume and capacity of the lung would be affected by body weight [26]. This may be the reason why the heart and lungs of LAEC mice are heavier than HAEC mice in this study (Table 2). Past studies showed that the intensity of intrinsic aerobic exercise capacity has significant effects on posture and metabolism. The body weight of low-capacity running (LCR) rats on a normal diet was significantly higher than that of high-capacity running (HCR) rats by 20% [27]. Unexpectedly, after only eating a high-fat diet for three days, the body weight of the LCR rats increased significantly, but there was no effect on the HCR rats [11]. During pregnancy, in order to ensure the nutrition of the fetus and breastfeeding, the metabolism of pregnant women changes, leading, especially, to an increase in body fat. Excessive weight gain during pregnancy can lead to long-term obesity after childbirth. Although the gestation period of mice is relatively short, pregnancy still significantly increases the fat content of pregnant mice [28]. In addition, after pregnancy and after weaning, and before intervention with the high-fat diet, the body weight of LAEC postpartum mice was nearly 10% higher than that of HAEC mice. Previous research reported that HFD-induced weight gain results from increased energy intake due to the high energy density of the HFD [29,30]. However, in this study, no increase in total energy consumption occurred with HFD feeding in either line. Thus, both lines compensated for the high energy density of the HFD by reducing the amount of food they consumed. Moreover, neither line differed in their body weight relative energy intake with the control diet (Table 1). Since the high-fat feed used in this study is a mixture of lard, fructose, and standard feed, it had lower water content and greater dryness than ND, and the HFD product is moist and thicker. Therefore, we inferred that HFD intake might contain higher water intake and reduced fluid intake, or ND might compensate for lower fluid intake in food through larger water intake [31]. However, it may also produce metabolic water by oxidizing adipose tissue and dietary fat. In addition, HFD intake could reduce kidney mass, changed kidney function, caused kidney damage, and differences in water and solute retention and excretion [32,33]. A past study found that rats using HFD drank less water than the control group and compensated by excreting thicker urine [34]. It is inferred from this that this may be a possible factor in our study, whether ingesting HFD in HAEC or LAEC mice, can significantly reduce kidney weight and reduce water intake (Table 1 and Table 2).

Although it is well-known that HFD intake is one of the key factors in increasing body weight and fat. Previously studies had pointed out that fat intake did not drastically stimulate fat oxidation or energy consumption but promoted fat storage [35]. Therefore, body obesity was positively correlated with dietary fat intake, but had nothing to do with the use of macronutrients or total food intake [36]. In our result, compared with HAEC, HFD intake on LAEC mice had more calorie efficiency to not only more increased body weight (Table 1), but also significantly increased the production and accumulation of fat and visceral fat. In terms of absolute and relative tissue weights, except for the significant increase in ovarian fat, adrenal fat, and mesenteric fat, the LAEC-HFD group mice had the heaviest liver tissue weight. A previous study indicated that intake of HFD would inhibit muscle growth, cause damage to muscle metabolism and reduce muscle mass; however, there are still many studies that hold the opposite view [37]. Ingestion would not only make it easier to accumulate in fat cells, but also might cause lipid accumulation in muscles, which in turn would increase muscle mass, but not lean body mass [38]. Past studies have also shown that LCR significantly increases skeletal muscle lipid metabolism disorders compared with HCR for the same high-fat dietary intake [39]. This seems to be more consistent with our research (Table 2). No matter if HAEC or LAEC mice ingested HFD, heavier muscle mass was found. From the tissue sections, we found that compared with ND intake, HFD intake significantly increased vacuoles in fat, especially in the LAEC-HFD group, where the effect was the most obvious. In addition, the most lipid droplets were found in the liver (Figure 7A,B). This seems to be related to fatty acid oxidation capacity (FAO), liver mitochondrial metabolism, and insulin susceptibility. During long-term exercise, a higher amount of adenosine triphosphate (ATP) production is required to maintain liver gluconeogenic flux. In addition, fatty acids are increased by the lipolysis of triglycerides stored in fat and are delivered to the liver at a higher rate. The liver generates ATP by increasing mitochondrial oxidation of fatty acids [40]. Therefore, the obvious difference in liver mitochondrial content and function between HCR and LCR is one of the main characteristics obtained by selective reproduction of high endurance exercise capacity and low endurance exercise capacity [18]. The total liver FAO capacity is controlled by a variety of factors, including carnitine palmitoyl transferases 1α (CPT-1α) enzyme activity, liver mitochondrial content or density, the rate-limiting step of long-chain fatty acids entering mitochondria, amongst others, including substrate supply and energy status [41]. An overall FAO elevation indicates an increased coupling of acetyl-coenzyme (CoA) flux derived from lipids produced by β oxidation through the tricarboxylic acid (TCA) cycle, away from potential pathological incomplete oxidation products [42]. A previous study found that, compared with highly adapted HCR rats, LCR rats showed lower liver mitochondrial content, leading to a decrease in FAO capacity, which in turn increased the risk of liver steatosis [43]. In addition, HCR rats mainly transport lipids to muscles, where they are eventually oxidized and cannot be released into the circulatory system again. Conversely, LCR rats mainly transport lipids in the diet to the fat pad, where the fat is stored for a period of time and is oxidized and metabolized in tissues such as the heart, muscle, and liver [44]. In the current study, although HFD intake increased the TC and TG levels in the liver and blood, compared with LAEC mice, HAEC mice showed better lipid metabolism and lipid protection (Figure 5 and Figure 6).

Systemic insulin resistance is thought to be associated with lipid accumulation in skeletal muscle [45]. When too many fatty acids enter the mitochondria, the increase in incomplete fatty acid oxidation can cause oxidative stress or other mitochondrion-derived metabolites, which can lead to insulin resistance [46]. Previous studies showed that HFD intake only significantly increases the glucose transport rate and glycogen synthesis rate in the skeletal muscle of HCR rats and protects against diet-induced insulin resistance. By contrast, LCR rats showed decreased insulin sensitivity after HFD feeding, which may be related to the large amount of intramuscular lipids in this strain [11]. Increased storage of lipid metabolites (glycerol, glyceryl-CoA, and ceramide) in muscle can impair the action of insulin [47]. In our research, we found that HAEC mice showed greater glucose tolerance than LAEC mice fed the high-fat diet (Figure 3). Increased fatty acid supply may lead to increased muscle β-oxidation, impairing muscle glucose use, and may cause obesity-associated insulin resistance in skeletal muscle [48,49]. The increased storage of lipids in insulin-sensitive tissues (such as skeletal muscle) was shown to be the main cause of decreased aerobic capacity, insulin resistance, and impaired glycogen synthesis [50]. It seems that this finding is confirmed in this research. Although excessive energy intake after being fed a high-fat diet will increase glycogen storage, compared with the LAEC mice, the HAEC mice still showed a higher glycogen storage capacity in both liver and muscle tissues (Figure 4). In our previous study, HAEC mice significantly upregulated miR-383, miR-107, miR-30b, miR-669m, miR-191, miR-218, and miR-224 compared with LAEC mice [51]. Among them, miR-191 and miR-224 are thought to reduce adipogenesis and inhibit synthesis by regulating acyl-CoA synthase activity [52]. In addition, miR-107 has the ability to regulate the gene expression of caveolin-1, an upstream regulator of insulin receptors, thereby improving insulin sensitivity [53]. These may be the mechanisms by which HEAC postpartum mice who ingested HFD in this study were able to obtain better lipid and metabolic protection. In this study, we preliminarily explored the intuitive differences in high-fat diet-induced lipogenesis and lipid metabolism in HAEC and LAEC postpartum mice and confirmed that HEAC postpartum mice had more advantages than LAEC postpartum mice. However, it is necessary to further explore the relevant mechanism of action in the future, and to further understand whether adding exercise training under innate conditions has a regulatory effect on lipid metabolism and protection.

## 5. Conclusions

In conclusion, postpartum poses a high risk to those consuming high-fat diets, which are more likely to cause fat accumulation, metabolic diseases, and decrease exercise performance, which agrees with the findings in our research. However, HAEC mice showed better protection against fat and metabolic diseases than LAEC mice. Our results suggest that postpartum mice with intrinsic a high aerobic exercise capacity are more resistant to obesity and metabolic disease induced by HFD, whereas low aerobic exercise capacity exacerbated HFD-induced complications.

## Figures and Tables

**Table 1:** Baseline exercise capacity, growth curve and dietary profiles during experiments.

Characteristic	HAEC-ND	HAEC-HFD	LAEC-ND	LAEC-HFD	*p* Value (Main Effect of)		
					AEC	HFD	Interaction (AEC × HFD)
Swimming capacity at 6th week of age (min)	163.67 ± 4.67 ^a^	164.00 ± 4.62 ^a^	4.22 ± 0.33 ^b^	4.32 ± 0.24 ^b^	<0.0001	0.7812	0.4901
Initial BW (g)	39.9 ± 1.0 ^a^	41.4 ± 1.2 ^a^	44.8 ± 0.8 ^b^	44.0 ± 0.9 ^b^	<0.0001	0.0902	0.2177
Final BW (g)	34.1 ± 0.6 ^a^	43.0 ± 1.5 ^b^	40.3 ± 0.7 ^b^	52.8 ± 3.2 ^c^	0.0003	<0.0001	0.4122
Water intake (mL/mouse/day)	9.35 ± 0.14 ^b^	4.42 ± 0.23 ^a^	9.10 ± 0.34 ^b^	4.09 ± 0.08 ^a^	0.2272	<0.0001	0.8269
Diet intake (g/mouse/day)	5.47 ± 0.07 ^c^	3.44 ± 0.08 ^a^	6.24 ± 0.10 ^d^	4.01 ± 0.07 ^b^	<0.0001	<0.0001	0.2039
Kcal intake (g/mouse/day)	18.22 ± 0.24 ^a^	17.67 ± 0.42 ^a^	20.92 ± 0.32 ^b^	20.57 ± 0.34 ^b^	<0.0001	0.1260	0.6079
Calorie efficiency (%)	−0.42 ± 0.16 ^a^	0.13 ± 0.27 ^b^	−0.28 ± 0.11 ^a^	0.52 ± 0.51 ^c^	0.1016	<0.0001	0.4306

The weight and diet were measured regularly for the 4 groups allocated as HAEC-ND, HAEC-HFD, LAEC-ND, and LAEC-HFD. All the data are presented as mean ± SEM and were analyzed by two-way ANOVA. Different superscript letters (a, b, c, d) in the same column indicate significant differences between groups, *p* < 0.05. ND, normal chow diet; HFD, high-fat diet; HAEC, high aerobic exercise capacity; LAEC, low aerobic exercise capacity.

**Table 2:** Intrinsic aerobic exercise capacity in HFD intake on body composition.

Characteristic	HAEC-ND	HAEC-HFD	LAEC-ND	LAEC-HFD	*p* Value (Main Effect of)		
					AEC	HFD	Interaction (AEC × HFD)
Liver (g)	0.54 ± 0.01 ^a^	0.68 ± 0.02 ^bc^	0.64 ± 0.03 ^ab^	0.78 ± 0.07 ^c^	0.0251	0.0011	0.9624
Kidney (g)	0.49 ± 0.03 ^ab^	0.42 ± 0.02 ^a^	0.58 ± 0.03 ^c^	0.50 ± 0.02 ^b^	0.0003	0.0057	0.478
Perirenal fat paid (g)	0.16 ± 0.03 ^a^	1.04 ± 0.13 ^b^	0.22 ± 0.03 ^a^	1.47 ± 0.30 ^b^	0.2262	<0.0001	0.2648
Mesenteric fat (g)	0.85 ± 0.05 ^a^	1.58 ± 0.12 ^b^	1.10 ± 0.06 ^a^	2.16 ± 0.29 ^c^	0.02	<0.0001	0.4103
Ovarian fat paid (g)	0.24 ± 0.03 ^a^	1.32 ± 0.21 ^b^	0.32 ± 0.04 ^a^	2.04 ± 0.40 ^c^	0.129	<0.0001	0.2059
Muscle (g)	0.35 ± 0.01 ^a^	0.40 ± 0.01 ^b^	0.37 ± 0.01 ^a^	0.40 ± 0.01 ^b^	0.5257	0.0006	0.2945
Heart (g)	0.21 ± 0.01 ^a^	0.21 ± 0.01 ^a^	0.25 ± 0.01 ^b^	0.24 ± 0.01 ^ab^	0.0131	0.5491	0.7435
Lung (g)	0.27 ± 0.01 ^a^	0.28 ± 0.01 ^a^	0.37 ± 0.03 ^b^	0.40 ± 0.02 ^b^	<0.001	0.371	0.5826
BAT (g)	0.08 ± 0.01 ^a^	0.09 ± 0.01 ^ab^	0.10 ± 0.01 ^b^	0.11 ± 0.01 ^b^	0.0057	0.135	0.4721
Liver (%)	1.47 ± 0.02 ^a^	1.86 ± 0.05 ^bc^	1.73 ± 0.09 ^ab^	2.13 ± 0.18 ^c^	0.0249	0.001	0.9488
Kidney (%)	1.35 ± 0.07 ^ab^	1.15 ± 0.05 ^a^	1.58 ± 0.08 ^c^	1.36 ± 0.05 ^b^	0.0003	0.006	0.4776
Perirenal fat paid (%)	0.45 ± 0.09 ^a^	2.83 ± 0.34 ^b^	0.61 ± 0.09 ^a^	4.01 ± 0.81 ^b^	0.227	<0.0001	0.2654
Mesenteric fat (%)	2.31 ± 0.13 ^a^	4.31 ± 0.34 ^b^	3.00 ± 0.15 ^a^	5.89 ± 0.80 ^c^	0.1286	<0.0001	0.2066
Ovarian fat paid (%)	0.64 ± 0.23 ^a^	3.60 ± 0.58 ^b^	0.86 ± 0.10 ^a^	5.55 ± 1.10 ^c^	0.1286	<0.0001	0.2066
Muscle (%)	0.96 ± 0.34 ^a^	1.10 ± 0.03 ^b^	1.02 ± 0.01 ^a^	1.09 ± 0.03 ^b^	0.5193	0.0005	0.2984
Heart (%)	0.58 ± 0.21 ^ab^	0.57 ± 0.02 ^a^	0.67 ± 0.03 ^b^	0.64 ± 0.03 ^ab^	0.0144	0.4989	0.7733
Lung (%)	0.73 ± 0.26 ^a^	0.76 ± 0.03 ^a^	1.00 ± 0.09 ^b^	1.09 ± 0.06 ^b^	<0.001	0.3692	0.5667
BAT (%)	0.21 ± 0.07 ^a^	0.25 ± 0.02 ^ab^	0.28 ± 0.02 ^b^	0.29 ± 0.02 ^b^	0.0144	0.1832	0.5193

Data are mean ± SEM for *n* = 8 mice in each group. Different superscript letters (a, b, c) in the same column indicate significant differences between groups, *p* < 0.05. by two-way ANOVA; PFD, perirenal fat paid; MT, mesenteric fat; OFP, ovarian fat pad; BAT: brown adipose tissue.

## Data Availability

The data presented in this study are available within the article.
